# Gut Microbiota as Potential Biomarker and/or Therapeutic Target to Improve the Management of Cancer: Focus on Colibactin-Producing *Escherichia coli* in Colorectal Cancer

**DOI:** 10.3390/cancers13092215

**Published:** 2021-05-05

**Authors:** Julie Veziant, Romain Villéger, Nicolas Barnich, Mathilde Bonnet

**Affiliations:** 1 Microbes Intestin Inflammation et Susceptibilité de l’Hôte (M2iSH) UMR 1071 Inserm/Université Clermont Auvergne, USC-INRAE 2018, CRNH Auvergne, 63000 Clermont-Ferrand, France; julieveziant@gmail.com (J.V.); romain.villeger@univ-poitiers.fr (R.V.); nicolas.barnich@uca.fr (N.B.); 2 Department of Digestive, Hepatobiliary and Endocrine Surgery Paris Descartes University Cochin Hospital, 75000 Paris, France; 3 Laboratoire Ecologie & Biologie des Interactions, UMR CNRS 7267 Université de Poitiers, 86000 Poitiers, France

**Keywords:** intestinal microbiota, CoPEC, colibactin, cancer, *E. coli*, dysbiosis, anti-cancer treatment, prognosis, biomarker, colorectal cancer

## Abstract

**Simple Summary:**

Gut microbiota is emerging as new diagnostic and prognostic marker and/or therapeutic target to improve the management of cancer. This review aims to summarize microbial signatures that have been associated with digestive and other cancers. We report the clinical relevance of these microbial markers to predict the response to cancer therapy. Among these biomarkers, colibactin-producing *E. coli* are prevalent in the colonic mucosa of patients with colorectal cancer and they promote colorectal carcinogenesis in several pre-clinical models. Here we discuss the promising use of colibactin-producing *E. coli* as a new predictive factor and a therapeutic target in colon cancer management.

**Abstract:**

The gut microbiota is crucial for physiological development and immunological homeostasis. Alterations of this microbial community called dysbiosis, have been associated with cancers such colorectal cancers (CRC). The pro-carcinogenic potential of this dysbiotic microbiota has been demonstrated in the colon. Recently the role of the microbiota in the efficacy of anti-tumor therapeutic strategies has been described in digestive cancers and in other cancers (e.g., melanoma and sarcoma). Different bacterial species seem to be implicated in these mechanisms: *F. nucleatum*, *B. fragilis,* and colibactin-associated *E. coli* (CoPEC). CoPEC bacteria are prevalent in the colonic mucosa of patients with CRC and they promote colorectal carcinogenesis in susceptible mouse models of CRC. In this review, we report preclinical and clinical data that suggest that CoPEC could be a new factor predictive of poor outcomes that could be used to improve cancer management. Moreover, we describe the possibility of using these bacteria as new therapeutic targets.

## 1. Introduction

**Figure 1:**
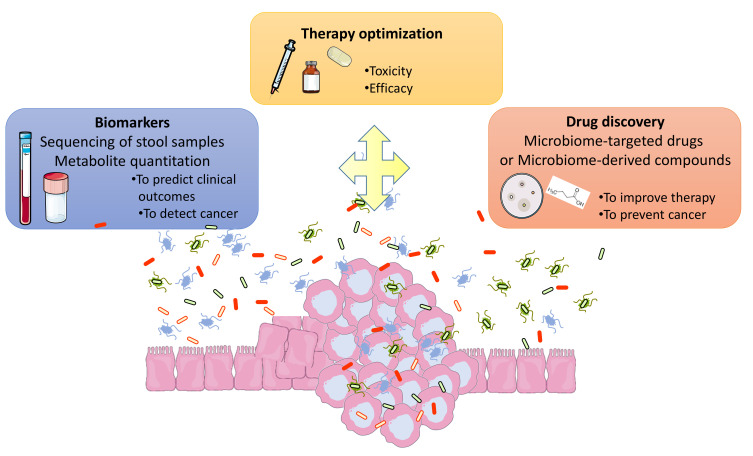
Intestinal microbiome for a better management of cancer patients. New biomarkers based on microbial composition of the stool are emerging to predict clinical outcomes. Metabolites signatures and/or sequencing oral samples will also be developed as diagnostic or prognostic biomarkers. Studies about interactions between gut microbiota and treatments (surgery, chemotherapy, immunotherapy, radiotherapy) could allow to improve their efficacy and decrease some side effects (e.g., post-surgical complications, toxicity). Gut microbiota data could be use for the discovery of new and innovative therapeutic tools as small bioactive molecule which mimic benefic microbial effect or targeting procarcinogenic bacteria. Microbial intervention could be developed including prebiotics, probiotics, phages. Natural products and/or diet complementation can also be considered. These strategies will have to be adapted according to tumor characteristics and to the patient’s environment, lifestyle, host susceptibility and comorbidities.

The human gut microbiota is a complex microbial ecosystem harboring more than 100 billion bacteria, archaea, viruses, parasites and fungi that maintain symbiotic interactions with the host [1,2,3]. This intricate relationship plays a crucial role in maintaining healthy homeostasis including nutrient absorption, vitamin biosynthesis, immune system development, the maintenance of epithelial mucosa integrity, and resistance against pathogens [4,5]. Given the symbiotic relationship between the host and gut microbiota, an alteration from the normal microbiota composition, referred to as dysbiosis, has been observed in several human diseases including digestive cancers and other extra-digestive malignancies [2,6,7]. Gut microbiota dysbiosis could promote carcinogenesis in various tumor pathologies [8]. Association between repeated courses of antibiotics and the development of a variety of tumors has been demonstrated [9]. Most evidence supporting a causal role for gut dysbiosis as a modulator of cancer development is related to colorectal cancer (CRC) [10,11,12,13]. Gut microbiota have also been associated with a number of other malignancies, including hepatocellular carcinoma and breast cancer via its influence on energy metabolism and obesity and other specific mechanisms (for review [8]). In addition, scientists have investigated the entire microbial community in the tumor landscape through next generation sequencing technologies [14,15]. Even if the signature of dysbiosis is not yet defined in cancers, one emerging translational application of the gut microbiome is its potential as diagnostic and/or prognostic biomarker or as a therapeutic target (cf. Figure 1). The intestinal microbiota could therefore be a new tool to improve the management of cancers. Because the colonic microbiota is mostly represented by bacteria, the bacterial community remains the most studied.

In this review, we provide clinical data on the potential use of gut microbiota-related biomarkers for cancer screening and prognosis. We describe the results obtained for CRC and for others cancers. We next focused on colibactin-associated *E. coli* (CoPEC), a pro-carcinogenic bacterium that is more prevalent in aggressive form of CRC [16,17]. We discussed the preclinical and clinical data, which suggest that CoPEC could be a new prognostic biomarker in CRC management. Finally, we describe the possibility of using CoPEC as a new therapeutic target.

## 2. Gut Microbiota Biomarker for Cancer Screening

### 2.1. Intestinal Microbiota Biomarker for CRC Screening

The actual 5-year survival rate is 90% for a localized CRC, but this rapidly decrease to <15% for metastatic disease [18]. For this reason, an early, noninvasive, and accessible method for CRC screening is critical. The main screening strategy used worldwide is the fecal immunochemical test (FIT), which has a limited sensitivity of 79% for CRC diagnosis [19]. The multitarget stool DNA test, not routinely used, is very effective in detecting CRC (sensitivity 92.3%) but presents a higher false-positive rate than FIT [20]. Therefore, more accurate and noninvasive biomarkers for early CRC screening is still needed. Given the link between gut microbiota dysbiosis and CRC [21], microbial markers have recently emerged as a promising indicator in early CRC screening (cf Table 1). Known specific oncogenic gut bacteria have been assessed individually from both fecal and mucosal samples from patients with CRC, including strains of *Bacteroides fragilis*, *Escherichia coli*, *Enterococcus faecalis*, *Streptococcus gallolyticus,* and *Fusobacterium nucleatum* [16,17,21,22,23,24,25]. Over the last few years, many studies using sequencing methods with whole-genome shotgun (WGS) and/or 16S RNAr DNA sequencing have also highlighted several signatures (mostly in feces) between individuals with CRC and healthy control subjects [13,26,27,28,29,30]. Overall, these datasets revealed a global compositional shift in microbiota from individuals with CRC compared to healthy controls, notably with a reduced bacterial [30,31,32]. No single specific oncomicrobe has been found to be universally present in all patients with CRC. Significantly enriched and depleted fecal microorganisms have been identified between CRC and control populations. Differences were not affected when controlling for potential confounding by age, body mass index (BMI) or sex (40). These identified polymicrobial signatures could potentially be harnessed as screening biomarkers for CRC [25,30,31,32,33,34,35,36,37] (cf. Table 1). However at present, these is no consensus to define this signature. Consistently, alone or combined with other bacteria, *Fusobacterium nucleatum* emerged as a fecal marker in CRC both in feces and tumor samples compared to control subjects and surrounding normal tissue [30,34,35,38]. In addition, quantifying fecal *Fusobacterium nucleatum* abundance could improve the performance of the FIT in detecting CRC and advanced adenoma with an increase in the AUC from 0.86 to 0.95 [27,39]. Overall, the majority of the published data were obtained from fecal sample analysis. Because oral and gut microbiomes were predictive of each other and over-representations of several oral bacteria as *F. nucleatum* were detected in the stool from patients with CRC, microbial-biomarkers of CRC could be also investigated in oral samples [33,40]. Recently, Dohlman et al. discovered promising endogenous microbial DNA signatures in blood samples from CRC patients featuring mucosal barrier injury in CRC [41].

Microbial-derived metabolite analyses in human biofluids (blood, urine, saliva, and fecal) could also be a promising and noninvasive approach for early CRC screening [42]. Although the method of sample collection is not standardized, four human studies supported the screening potential of fecal metabolic profiling using the nuclear magnetic resonance (NMR) method in CRC cohort patients [43,44,45,46]. Fecal metabolite levels such as lactate, glucose, and some amino acids were higher in CRC patients than in controls [43,45]. Moreover, the levels of SCFAs, glutamate and succinate varied significantly according to CRC stage [45]. In a recent study, Lin et al. demonstrated that fecal acetate had the highest diagnostic performance for discriminating CRC from controls [44]. Conversely, Chen et al. observed that butyrate and several butyrate-producing bacteria were depleted in CRC patients stools [47].

Extended validation studies are required to define CRC signatures of dysbiosis. Future investigations should consider the heterogeneity of CRC. Because microbiota differ with gender and life style, these parameters have to be considered [1,32]. Moreover different clinical studies have suggested that the dysbiotic signature of CRC might be different according to the tumor characteristics (KRAS, BRAF mutations, and DNA mismatch repair MMR alterations) and/or tumor sidedness (proximal or distal location) [57,58,59,60,61,62,63,64].

### 2.2. Intestinal Microbiota Biomarker for Other Cancers Screening

While this has been relatively well studied in CRC, there are also emerging data suggesting gut microbiota dysbiosis as a potential noninvasive tool for early diagnosis of a variety of cancers (cf. Table 1). Even if 70% of gastric cancer (GC) is associated with *Helicobacter pylori* infection, this bacterium is not a relevant screening marker. Indeed, only 1–4% of *H. pylori* infected individuals will eventually develop GC. Recently, two clinical studies explored the correlation between fecal bacteria markers and GC diagnosis/occurrence compared to healthy people [48,49]. Liu et al. interestingly identified *Desulfovibrio*, *Escherichia*, *Faecalibacterium*, or *Oscillospira* as fecal biomarkers to predict GC with a precision of 90% or above [48]. Moreover, it has been suggested that the gut microbiota could play a role in the carcinogenesis of hepatocellular carcinoma (HCC) through the gut–liver axis [65,66]. Ren et al. [50] collected 419 human samples and found that fecal microbial diversity was increased in early HCC versus cirrhosis. A core set of 30 OTUs was sufficient to identify early HCC. Moreover, clinical studies of oral, fecal, and pancreatic microbiota composition have been reported in relation to pancreatic ductal adenocarcinoma (PDAC) [52,67,68]. Researchers have attempted to identify the fecal microbiota of patients with PDAC when compared to control patients. At the phylum level, a significant increase in Bacteroidetes abundance and a decrease in Firmicutes and Proteobacteria abundances were seen in the fecal microbiota of patients with PDAC versus healthy controls [51]. A recent clinical study provided sequencing data on the gut microbiota composition of esophageal cancer (EC) patients and screened out the optimal potential microbiota biomarkers for EC screening. The authors found that *Lachnospira* had higher accuracy in EC diagnosis [53].

In addition to these studies on digestive tract cancers, intestinal microbiota dysbiosis has been described in patients with cancer in other locations. Growing evidence has demonstrated a gut microbiota imbalance in breast cancer (BC) [69]. Several studies identified a variable gut microbiota composition in women with BC with elevated levels of Clostridiaceae, *Faecalibacterium*, and Ruminococcaceae, and a decrease in the levels of *Dorea* and Lachnospiraceae compared to paired healthy controls [54], confirming that the gut microbiome could be used as a relevant biomarker in BC investigation. Zhu et al. used shotgun metagenomic analysis and showed that postmenopausal women with BC have a different fecal microbiota composition than healthy controls [55]. Moreover, although the role of the lung microbiome in lung cancer (LC) has been investigated, very few studies have evaluated the gut microbiota involvement in LC development. Zhuang et al. [56] compared the gut microbiota of 30 LC patients to 30 healthy controls and found a higher abundance of the bacterial phylum Actinobacteria and genus *Bifidobacterium* in controls, while patients with LC showed elevated levels of *Enterococcus*, suggesting their use as possible biomarkers of LC.

Currently, intestinal microbiome biomarkers at the metagenomic or metabolomics level are still not being used in clinical practice for cancer screening. Further prospective investigations are required to confirm whether these microbial biomarkers can successfully identify patients with an increased risk of cancer and provide early intervention.

## 3. Gut Microbiota Biomarkers Predicting Prognosis and/or Treatment Response

### 3.1. Biomarker to Predict Cancer Therapy Efficacy

Currently, an emerging approach is to consider the influence of the gut microbiota on cancer therapy efficacy, thereby raising the possibility of using gut microbial community as a predictive biomarker for cancer treatments. Evidence from recent preclinical and clinical studies suggests that the gut microbiota may influence the efficacy of antitumor therapeutic strategies, especially immunotherapy and chemotherapy, as well as radiotherapy and surgery. Specific mechanisms have been reported for digestive cancers, but also for other cancer, mostly through an integration of metagenomic shotgun sequencing and 16S rRNA gene sequencing of patient stool samples [70]. Herein, we review the clinical relevance of observational patient cohorts supporting the use of gut bacteria related biomarkers to predict the response to cancer therapy.

#### 3.1.1. Immunotherapy

A prime example of the gut microbiota influencing therapeutic responses is through immunotherapy. Over the last few years, immunotherapy has revolutionized the therapeutic approach to cancer. Immune checkpoint inhibitors (ICIs), including anti-PD-1 and anti-CTLA-4 have become the most promising treatment, with prolonged responses and improved survival in both solid tumors (for example: melanoma and renal cell carcinoma, non-small cell lung cancer, and mismatch-repair deficient colorectal cancer) and lymphomas [71,72,73,74]. However, immunotherapy only benefits a subset of patients. Preclinical mouse models showed that the gut microbiota modulates tumor response to ICIs [75,76]. Human gut metagenomic analysis revealed that responder patients had different microbiota compositions than non-responders. For example, Chaput et al. [77] revealed, in a cohort of 26 patients with metastatic melanoma treated with ipilimumab (anti-CTLA-4), that patients whose baseline gut microbiota was enriched with *Faecalibacterium* and other Firmicutes had longer progression-free survival and overall survival. A recent clinical cohort reported that antibiotic administration could inhibit the clinical benefit of anti-PD-1 in patients with metastatic renal or lung cancer. The authors also revealed correlations between clinical responses to anti-PD-1 and the relative fecal abundance of *Akkermansia muciniphila* in patients at the time of diagnosis [72]. Moreover, in 2018, two studies published in *Science* confirmed that the gut microbiota composition influences clinical response to anti-PD-1 therapy in melanoma patients [71,73]. Gopalakrishnan et al. [71] used the metagenomics WGS approach on stool sample from 43 metastatic melanoma patients to observe that *Fecalibacterium* spp. were overrepresented in responder patients while *Bacteroides thetaiotaomicron*, *Escherichia coli*, and *Anaerotruncus colihominis* were enriched in non-responders. Similarly, Matson et al. [73] showed that bacterial species such as *Bifidobacterium longum*, *Collinsella aerofaciens*, and *Enterococcus faecium* were more abundant in the feces from responder patients than non-responders. Both studies demonstrated that the « responder » phenotype could be transferred into germ-free or antibiotic-treated mouse models via fecal microbiota transplant (FMT), leading to a greater efficacy of ICI therapy while FMT from the non-responders profile failed to. More recently, Baruch et al. and Davar et al. provided the first-in-human proof-of-concept evidence for the effectiveness of FMT to affect immunotherapy response in metastatic melanoma patients [78,79]. Based on these published reports, the clinical relevance of the gut microbiota as a novel biomarker of ICI response must be validated in additional prospective clinical studies.

#### 3.1.2. Chemotherapy-Radiotherapy

The Gut microbiota may shape responses to chemo and/or radiotherapy. Alexander et al. have proposed in their review the ‘TIMER’ mechanistic framework (from Translocation, Immunomodulation, Metabolism, Enzymatic degradation, and Reduced diversity and ecological variation) to explain how gut bacteria influence chemotherapy effects on the host [80]. Numerous preclinical studies have demonstrated the involvement of the gut microbiome in the efficacy of a range of chemotherapies such as cyclophosphamide [81], oxaliplatin [74,82] or gemcitabine [83] in CRC and other cancer types. More recently, a metagenome association study in lung cancer patients showed that *Streptococcus mutans* and *Enterococcus casseliflavus* were linked to better chemotherapy outcomes [84]. Clinical evidence-based research on the role of potential gut microbial biomarkers for predicting patient’s response to chemotherapy is scarce. In 2020, two Asian cohort studies [85,86] aimed to evaluate the predictive value of the gut microbiome in terms of the response after preoperative concurrent chemoradiation (nCCRT) in patients with rectal cancer. Jang et al. [85], after analyzing 45 fecal samples, showed that Bacteroidales (Bacteroidaceae, Rikenellaceae, *Bacteroides*) were relatively more abundant in patients with a noncomplete response than in those with complete response. Additionally, the authors found that *Duodenibacillus massiliensis* was associated with a complete response. Yi et al. [86] identified potential microbial biomarkers for predicting the response to nCCRT in locally advanced rectal cancer: butyrate-producing bacteria, including *Roseburia*, *Dorea*, and *Anaerostipes* were overrepresented in responders, whereas *Coriobacteriaceae* and *Fusobacterium* were markedly higher in non-responders. Although gut dysbiosis could be a relevant biomarker to predict radiotherapy-induced mucositis [87,88], the direct impact on the patients’ response to radiotherapy has not yet been investigated yet. Recently Guo et al. described in mice that radiation survivors could be identified by specific bacterial and metabolite profiles (Lachnosspiraceae and Enterococcaceae increases and downstream metabolites such as propionate and tryptophan pathways) [89]. These data suggest that some microbiota-associated markers could be identified to predict radiation injury. Further clinical studies are needed to identify the microbial populations involved in radioresistance.

#### 3.1.3. Surgery

To the best of our knowledge, a key role of gut microbiota in surgical outcomes has mainly been demonstrated in CRC. In particular, anastomotic leaks (AL) constitute the most life-threatening postoperative complications following colorectal procedures (ranging from 2 to 19%) despite a consistent improvement of surgical techniques and perioperative care [90]. The potential role of gut microbiota on the pathogenesis of AL following CRC surgery is increasingly obvious [91,92]. Previous works on animal models have suggested the causal role of specific microorganisms presenting with high collagenolytic properties including *Enterococcus faecalis* and *Pseudomonas aeruginosa* [93]. These specific bacterial strains possess high collagenase activity and activate matrix metalloproteinase (MMP-9), thus leading to tissue breakdown and AL through collagen-degrading ability. Shogan et al., found in humans undergoing colonic resection that their anastomotic tissues were still colonized with *E. faecalis* and other bacterial strains with collagen-degrading/MMP-9-activating properties. However, the available literature reporting the relationship between gut microbiota and AL in human patients with CRC is very scarce. The presence of *E. faecalis* in the drain fluid of patients with colorectal AL suggests its potential use as a fast screening tool for AL prevention in the early postoperative phase [94]. Based on data from a pilot study [95], Van Praagh et al. investigated the mucosa-associated microbiota composition in AL depending on the placement (or not) of a bioresorbable sheath (C-seal) into the anastomosis [96]. In non-C-seal patients, AL was significantly associated with a lower microbial diversity and a higher abundance of the mucin-degrading microbiome families *Lachnospiraceae* and *Bacteroidaceae* compared to matched patients with no AL. The authors indicated that, in non-C-seal patients, a gut microbiota composition consisting of ≥60% *Lachnospiraceae* and *Bacteroidaceae* was a predictive factor for AL. More recently, Palmisano et al. [97] demonstrated an increase in preoperative aggressive bacteria *Acinetobacter lwoffii* and *Hafnia alvei* and a low abundances of protective bacteria in five patients with AL after colonic resection. All of this clinical evidence strengthened the hypothesis that a peculiar gut microbiota composition could represent a risk factor for AL occurrence and could be a good biomarkers to predict AL. Surgical site infection (SSI: superficial, deep, or organ/space) and postoperative ileus (POI) are other common complications following surgical resection of CRC. Currently, very few clinical studies have linked gut microbiota with SSI or POI-related bacteria in CRC patients after surgery. Only one recent clinical study reported *Faecalibacterium* in mucosal samples from CRC patients as a possible predictive biomarker of POI [98]. Ohigashi et al. is the only clinical report to identify causative fecal bacteria enriched in SSI after CRC surgery: *S. aureus*, *P. aeruginosa*, and *Enterococcus* spp. [99]. Altogether, further clinical investigations to identify microbial signatures associated with a high risk of postoperative complications following colorectal surgery are needed. Prospective clinical studies are ongoing including our METABIOTE study [100].

### 3.2. Biomarker Predicting Prognosis and Long-Term Outcomes in CRC

Several studies have also demonstrated that specific microbial markers may serve as a prognostic indicator of cancer survival especially in CRC. The most studied bacteria related to CRC’ patient survival is *Fusobacterium nucleatum*. Flanagan et al. found a shortened disease-free survival in CRC patients related to higher levels of *F. nucleatum* [101]. The mucosal amount of *F. nucleatum* was associated with an advanced stage, proximal tumor location and shorter patient survival by Mima et al. [63]. In addition, a high abundance of *F. nucleatum* was associated with molecular alterations such as CIMP high, wild-type p53, MSI-high and BRAF mutation in colon tumor tissue [102]. The presence of enterotoxigenic *Bacteroides fragilis* in the colonic mucosa was also associated with a higher CRC stage. Wei and coworkers [102] concluded that the abundance of *F. nucleatum* or *B. fragilis* was a prognostic biomarker of poor survival. Moreover, Bonnet et al. [16] observed an increased level of mucosa-associated and internalized *E. coli* in CRC compared to normal tissue patients. A relationship between poor CRC prognostic indicators (tumor-node-metastasis stage) and colonization of mucosa by *E. coli* was reported. Pathogenic *E. coli* strains producing a genotoxin named « colibactin » (CoPEC) were more prevalent in the mucosa of CRC patients with stages III/IV than stage I tumors. More validations studies are needed before using these biomarkers as prognosis factors in daily clinical practice.

In conclusion, preclinical tumor models and observational cohorts of cancer patients are providing accumulating evidence that the composition of the gut microbiota seems to play a decisive role in the response of cancer patients to anticancer therapeutics such as chemotherapy, radiotherapy or ICIs. These studies mostly used high-throughput bacterial 16S rRNA gene sequencing and metagenomics approaches but using metabolome analysis in this context appears to be a promising method. Future challenges should be to develop interventional approaches by modulating the gut microbiota composition to safely boost the clinical efficacy of these anticancer treatments [103,104]. This new therapeutic strategy named “oncobiotic” is expected to strengthen the oncological arsenal [105,106,107]. Different strategies is developing ad oral administration of live microorganisms (bacteria and/or phages), probiotic, prebiotic, fecal microbiota transfer, or bacterial metabolites. They will be tested alone or in association with classical treatment and be adapted and personalized to the gut dysbiosis determined for each patient [105]. Further clinical research is needed to confirm the use of oncobiotics to target the microbiome and improve cancer treatment.

## 4. Colibactin-Producing *E. coli* in the Intestinal Microbiota

Colibactin is a bacterial genotoxin and was first identified in 2006 by Nougayrède et al. in the *E. coli* meningitis strain IHE3034 [108]. Colibactin-producing *E. coli* (CoPEC) has been isolated from the intestinal microbiota as commensal bacteria [109,110,111,112,113], in infectious diseases such as septicemia [110,114], newborn meningitis [112], urinary tract infections [106], and colorectal neoplasia tissues [22,115].

Several studies found that CoPEC was more common in the gut microbiota of 55 to 67% of CRC patients, while they were found in 18 to 21% of healthy subjects [17,22,115,116]. A study in Japan did not find an increased prevalence of *pks* genes in CRC patients (43%) when compared to healthy patients (46%) [117], but in their study, bacterial DNA was isolated by colonic lavage followed by PCR amplification, as opposed to the direct isolation of mucosa-associated *E. coli* from healthy and CRC patients as reported previously. Recently, a study by Iyadorai et al., reporting lower levels of colonization, confirmed this higher prevalence of CoPEC in CRC patient mucosa [118]. In addition, it was demonstrated that genes in the *pks* island were significantly more prevalent in tissue than blood, suggesting that *E. coli* strains expressing this gene are associated with CRC tissues [41]. Our team also reported that colonization of the intestinal mucosa by CoPEC was more prevalent in patients with stage III or IV CRC than in those with stage I CRC, suggesting a possible link with cancer aggressiveness [16]. In this study, we showed that the sex and the age of the patients with colon cancer did not influence the colonization by associated and internalized *E. coli*. Watanabe et al. observed that the prevalence of CoPEC was positively associated with male sex in healthy Japanese individuals [119]. The correlation between the presence of CoPEC and colorectal tumors has led to intensive research to unveil the possible pro-carcinogenic mechanisms of colibactin.

### 4.1. Pks Genomic Island and Colibactin Structure

Colibactin is synthesized from the *pks* genomic island which is a 54-kb nonribosomal peptide synthetase–polyketide synthase (NRPS–PKS) gene cluster that encodes the enzymes responsible for its biosynthesis [120]. Colibactin is a secondary metabolite that undergoes a prodrug activation mechanism. It involves the installation of a structural motif at the *N*-terminus, which is removed at the final stage of biosynthesis [121,122]. Notably, the *pks* island was identified in extraintestinal pathogenic *E. coli* but has also been found in other members of the family Enterobacteriaceae (*Klebsiella pneumoniae*, *Enterobacter aerogenes*, and *Citrobacter koseri*). The *pks* genomic island phylogeny indicated horizontal acquisition/transmission and the possibility of exchange between compatible *E. coli* subtypes [123].

Because of its high instability, the structure of colibactin has remained unknown for a long time. Since colibactin cannot be purified directly from a CoPEC culture media, a commonly employed strategy is to compare bacterial metabolites in *pks^+^-E. coli* and *pks*^−^-*E. coli* deficient for colibactin production. In *pks^−^-E. coli*, precolibactins accumulate, allowing their identification by mass spectrometry. However, the purification and characterization of precolibactins remains a challenge, and the compounds isolated so far have been obtained in extremely small quantities. The structure of colibactin has only been revealed recently. Since it was demonstrated that colibactin-producing bacteria cross-link DNA, the group of Crawford and colleagues used DNA as a probe to isolate colibactin from bacterial cultures and elucidated the structure of the colibactin residue when bound to two nucleobases [124]. Colibactin biosynthesis is not described in this paper since our team and others have extensively reviewed the process previously [125,126].

### 4.2. Pro-Carcinogenic Activity of CoPEC in CRC

**Figure 2:**
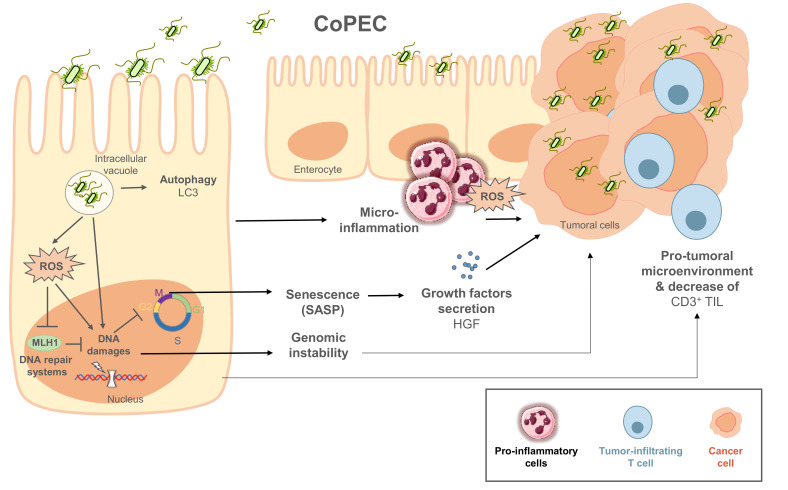
Pro-carcinogenic activity of CoPEC in colonic mucosa. Escape of CoPEC from autophagy in infected epithelial cells could lead colibactin to alkylate DNA, and further to cause DNA damage and then cell cycle arrest. In addition, CoPEC induces cellular oxidative stress, leading to inhibition of the DNA repair protein MLH1. All of these mechanisms participate in genomic instability in infected epithelial cells. In addition, CoPEC induced senescence of infected epithelial cells, accompanied by secretion of inflammatory mediators and growth, promoting the proliferation of adjacent uninfected cells. They could also affect the tumor immune microenvironment, even at distances from the tumor site through the reduction of tumor-infiltrating CD3^+^ T-cells.

Colibactin has been reported to cause DNA double-strand breaks in vitro [108]. Since this first publication, several teams have reported the induction of DNA double-strand breaks, in vitro and in vivo, as well as chromosomal instability through ROS production and cell cycle arrest, suggesting the potential role of colibactin in CRC development [22,61,108,113,127,128,129,130]. The pro-carcinogenic activity of CoPEC has been demonstrated in several preclinical animal models. CoPEC promoted colorectal cancer formation in multiple intestinal neoplasia (Apc^Min/+^) mice [16], in AOM-DSS-treated mice [131], in IL-10-deficient (IL-10^−/−^) mice treated with AOM [22], and in Apc^min/+/^IL-10^−/−^ mice [132]. Recently, we showed that autophagy in infected epithelial cell could control the pro-carcinogenic activity of CoPEC [133]. However, the precise mechanisms of colibactin pro-carcinogenic activity is still under investigation (cf. Figure 2).

Current data support a model in which colibactin alkylates DNA via a cyclopropane ring conjugated to α,β-unsaturated imine, leading to the formation of adenine-colibactin adducts and DNA crosslinks [124,134]. The induction of double-strand breaks has been reported to involve a copper-mediated oxidative cleavage [135]. In addition to DNA damage, CoPEC induces cellular oxidative stress in vitro, leading to decreased expression of DNA repair proteins MLH1 and MSH2, and thus increased genomic instability [61]. Recently, important studies have provided strong evidence regarding the etiological role of colibactin in the first step of human colorectal carcinogenesis [130,136,137]. Dziubańska-Kusibab et al. reported that colibactin-induced DNA double-strand breaks were enriched for an AT-rich hexameric sequence motif associated with distinct DNA-shape characteristics. The exact double-strand-break loci corresponded with mutational hot spots in previously identified cancer genomes [136,138]. In a second work, Iftekhar et al. investigated the transformation potential of a short-term infection with CoPEC using primary murine colon epithelial cells [130]. Infected organoids show characteristics of CRC cells (e.g., increased proliferation, impaired differentiation, several mutations in genes related to p53-signalling pathways). Finally, Pleguezuelos-Manzano et al. identified a unique mutational signature caused by exposure to CoPEC that is enriched in human CRC tumors and metastases [137]. After chronic exposure of human organoid cultures to human CRC-derived CoPEC or the corresponding colibactin-deficient *E. coli* strain, the authors used WGS on the organoids to show an increase in single-base substitutions and single T deletions at T homopolymers in colibactin-exposed cultures when compared to the mutant. After examination of the WGS datasets, the same mutational signature was detected in a subset of 5876 human cancer genomes from two independent cohorts, predominantly in CRC. Moreover, both mutational signatures were positively correlated with each other, suggesting their common origin. Therefore, to link colibactin-mutational signatures with oncogenic mutations, Clevers and his team showed that colibactin signatures were found in 2.4% of CRC driver mutations, with 5.3% of *APC* mutations having the highest rate. This molecular fingerprint provides a direct link between colibactin exposure and the DNA damage patterns that drive CRC development [139].

In addition to its genotoxic effect, it was demonstrated that CoPEC infection induces senescence of infected epithelial cells accompanied by the secretion of inflammatory mediators and growth factors (senescence associated secretory phenotype profile-SASP), thus promoting the proliferation of adjacent uninfected cells [131]. Dalmasso et al. demonstrated that cellular senescence was the consequence of the induction of miR-20a-5p expression, which targets SENP1, and leads to alteration of p53 SUMOylation [140]. In addition, CoPEC could also affect the tumor immune microenvironment, even distant from the tumor site. Indeed, we observed in both CRC patients and Apc*^Min/+^* mice colonized by CoPEC a reduced density of tumor-infiltrating CD3^+^ T-cells [141]. We noticed a significant decrease in antitumor T-cells in the MLNs of CoPEC-infected mice when compared to controls, suggesting that CoPEC could induce a pro-carcinogenic immune microenvironment through a reduction of the antitumor immune system. To our knowledge, the pro-carcinogenic effect of CoPEC has only been studied in CRC. However, the mechanisms described above, for which mediators act at distances from sites of infection, suggest that they could play a promoting role in other types of cancers. In addition to the role of CoPECs in colorectal carcinogenesis, different studies suggest that these bacteria may be diagnostic and prognostic factors in CRC patients.

### 4.3. CoPEC Detection as a Diagnostic or Prognostic Marker for Colorectal Cancers

Preclinical and clinical studies described that CoPEC has been associated with aggressive CRC and could be a poor prognostic factor. Specifically, a higher *E. coli* colonization rate and a higher prevalence of CoPEC are found in patients with TNM stage III or IV tumors (UICC TNM Classification, 8th Edition, 2017) [16]. Eklof et al. showed that the prevalence of CoPEC was progressively increased in the adenoma-carcinoma sequence [17]. They described that the presence of the *pks* island and *F. nucleatum* detection could predict cancer with a specificity of 63.1% and a sensitivity of 84.6%, suggesting the potential value of these microbial parameters for CRC diagnosis. In addition, we observed that CoPEC was more frequently identified in the microsatellite stable (MSS) CRC phenotype than the MSI (microsatellite instability) phenotype which is associated with better long-term outcomes [61]. Moreover, CoPEC gut colonization might contribute to modulating the immunotherapy efficacy in a preclinical model [141]. Indeed, our study showed that colonization by CoPEC led to a decreased response to anti-PD-1 immunotherapy in MC38-grafted mice. This work is the first to show that CoPEC can influence treatment efficacy. All of these data suggest that the presence of CoPEC in the intestinal microbiota could be a marker of poor prognosis of patients with CRC. In a clinical study, a high level of *E. coli* was shown in the feces of non-responders versus responders to anti PD-1 treatment of melanoma patients suggesting that *E. coli* and CoPEC could be predictive markers of cancers outcome [71]. Two clinical studies are in progress to evaluate the prognostic value of CoPEC in CRC and rectal cancer. First, the METABIOTE project evaluates the impact of new prognostic tools such as the gut microbiota, including the CoPEC prevalence and body composition profile, on surgical and oncologic results in a prospective cohort of 300 patients ([100]; ClinicalTrials.gov Identifier: NCT03843905). Second, the MICARE project aims to determine the CoPEC predictive value of a poor response to neoadjuvant treatment of rectal Cancers (ClinicalTrials.gov Identifier: NCT04103567). Extended studies are also required to determine the possible interaction between CoPEC and anticancer therapies in CRC and other cancers.

## 5. Targeting CoPEC in CRC Therapy

In the field of targeting gut microbiota for colon cancer therapy, two objectives have been distinguished: the first is the anti-tumor effect reducing activity of the pro-carcinogenic microorganisms, while another aspect is the increase of the CRC treatment efficacy eliminating the targeted bacteria. In regard to CoPEC targeting, most of the publications have focused on its anti-tumor effect.

Several strategies have been applied to target colibactin and inhibit its activity. Using docking approach, our team has discovered in 2016 two boron-based compounds that are ligands of the serine peptidase ClbP and prevent the genotoxicity induced by CoPEC in a preclinical mouse model [128]. Extensive additional data regarding the specificity of these compounds and possible side effects on host health are required. Oswald and his team, which first reported the existence of colibactin, reported that high iron intake could decrease the synthesis of colibactin [142,143], suggesting that iron could be a good candidate to regulate CoPEC virulence and carcinogenic factors. However, this observation suggests the complexity of targeting the intake of an essential nutrient with a controversial role in carcinogenesis. They also discovered a new interplay between the synthesis of the genotoxin colibactin and the polyamine spermidine by showing that endogenous spermidine synthase SpeE is required for full genotoxic activity of CoPEC [144]. Since polyamines are abundant in cancer tissue and are associated with cell proliferation, the relationship between spermidine levels in the colonic lumen and the pro-carcinogenic activity of CoPEC has to be investigated in order to find a possible way to inhibit a possible deleterious synergy in patients. The same team recently discovered that polyphosphate kinase (PPK) is required for the promoter activity of *clbB*, one of the genes in the *pks* island, and therefore for the production of colibactin [145]. They demonstrated that 5-aminosalicylic acid, a commonly prescribed drug and inhibitor of PPK, can inhibit the activity of the *clbB* promoter and thus reduce colibactin production, leading to reduced genotoxic activity in HeLa cells. In addition, all these strategies developed to eliminate CoPEC colonization and/or activity could improve the efficacy of CRC therapies. We have recently demonstrated in a preclinical model that gut colonization with a CoPEC strain could strongly reduce the efficiency of anti-PD-1 therapy [141]. Extensive studies are required to explore the role of CoPEC in cancer treatment outcomes and to find new strategies to target their activities to improve therapy efficiency.

The addition of natural compounds to the diet could appear to be an innovative alternative to reduce CoPEC activity. Decreased expression of *clbB* gene in the presence of the Sub-MIC concentrations of cinnamon essential oil and cinnamaldehyde has been observed in CoPEC isolated from CRC compared to untreated isolates [146]. Extracts of *Terminalia catappa*, *Psidium guajava,* and *Sandoricum koetjape*, as well as their metabolites tannin and quercetin, have been demonstrated to downregulate the expression of several *clb* genes in a CoPEC strain and protect eukaryotic epithelial cells from infection and DNA damage in vitro [147]. In a clinical study performed on healthy Japanese individuals, the prevalence of CoPEC was negatively associated with the intake of green tea [119]. Recently, impact of prebiotics (inulin and galacto-saccharide) on the genotoxicity of CoPEC, was studied in vitro. Unexpected results were obtained with an increase of DNA damage induced by the CoPEC after the treatment [148]. Preclinical studies are needed to confirm these data. Even if probiotic and/or an oncobiotic strategy has not yet been evaluated in vivo to reduce CoPEC colonization and/or activity, it could be an interesting approach in combination with CRC reference therapies.

These works highlight several promising approaches to reduce CoPEC activity in CRC patients harboring these bacteria and achieve promising outcomes in patient therapy. Preclinical and clinical studies will be necessary to develop and validate these innovative therapies targeting the CoPEC. With the current data, it is likely that the first clinical application of CoPEC detection will be the search for these bacteria in the feces as predictive biomarkers in order to better monitor CRC patients after treatment and prevent recurrences.

## 6. Conclusions

Among the multiple factors that may eventually result in carcinogenesis, gut microbiota dysbiosis has been described with increased attention. The gut microbiota could then lead to innovative and curative medical proposals for CRC and other cancers in addition to existing methods. Among these microbial factors, CoPEC emerges as an effective therapeutic target and predictive marker for CRC prognosis and long-term outcomes.

## Figures and Tables

**Table 1:** Intestinal microbiota-related biomarkers for the screening of CRC, digestive cancers and other cancers.

Cancer	Microbiota-Related Marker	Techniques	Samples	Ref
**CRC**	**Diversity**			
	Reduction of diversity (metagenome)	Shotgun metagenomic analysis	Feces	[30,31,32]
	Increase of diversity		Blood	[41]
**Polymicrobial signatures**				
	4 validated markers including *F. nucleatum, P. micra, S. moorei, P. stomatis*	Shotgun metagenomic analysis	Feces	[30]
	7 species including *B. fragilis, F. nucleatum, Porphyromonasasaccharolytica, P. micra, P. intermedia, A. finegoldii, and T. acidaminovorans*		Feces	[31]
	29 species including *F. nucleatum, P. micra, S. moorei, P. stomatis, Ruminococcus torques*		Feces	[35]
	16 species including *P. stomatis, F. nucleatum, Parvimonasspp., P. asaccharolytica, G. morbillorum, C. symbiosum and P. micra*		Feces	[37]
	22 species including *F. nucleatum* and *B. fragilis*	Shotgun metagenomic analysis and 16S rRNA gene sequencing	Feces	[34]
	34 species including *F. nucleatum, P. micra, S. moorei, P. stomatis*	16S rRNA gene sequencing	Feces	[33]
	12 species including *F. nucleatum, B. fragilis, P. micra, P. stomatis*		Tissue	[36]
	**Single bacteria**			
	*F. nucleatum (metagenome, 16S rRNA gene sequencing, PCR)*	Shotgun metagenomic analysis, 16S rRNA gene sequencing, PCR	Feces and Tissue	[25,30,35,36,41]
	*Colibactin-Producing E. coli (metagenome, PCR)*	Shotgun metagenomic analysis, PCR	Feces and Tissue	[16,17,22,25,35,41]
	*Bacteroidetes fragilis (ETBF) (metagenome, PCR)*	Shotgun metagenomic analysis, PCR	Feces and Tissue	[21,32,36]
	*Enterococcus faecalis (PCR)*	PCR	Feces	[23]
	*Streptococcus gallolyticus (metagenome, PCR)*	Shotgun metagenomic analysis, PCR	Tissue	[24,37]
	*Parvimonas micra (PCR)*	PCR	Feces	[25]
**Other digestive cancers**				
Gastric cancer	*Desulfovibrio, Escherichia, Faecalibacterium or Oscillospira*	16S rRNA gene sequencing	Feces	[48]
	*Lactobacillus* and *Megasphaera*		Feces	[49]
Hepatocellular Carcinoma	Increase of diversity (vs. cirrhosis) 30 OTUs including *Klebsiella, Prevotella* and *Haemophilus* (vs. control)	16S rRNA gene sequencing	Feces	[50]
Pancreatic ductal adenocarcinoma	Decrease of diversity	16S rRNA gene sequencing	Feces	[51]
	Increase of Bacteroidetes and decrease of Firmicutes and Proteobacteria. Set of 40 genera		Feces	[51]
	Set of 14 species including *Akkermansia* and *Bacteroidales*		Feces	[52]
Esophageal cancer	*Bacteroides, Bifidobacterium, Streptococcus, or Lachnospira*	16S rRNA gene sequencing	Feces	[53]
**Other cancers**				
Breast cancer	Increase of Clostridiaceae, *Faecalibacterium*, and Ruminococcaceae, and decrease of *Dorea* and Lachnospiraceae	16S rRNA gene sequencing	Feces	[54]
Breast cancer (post-menopausal)	14 optimal species markers including *Escherichia coli, Eubacterium eligens, Proteus mirabilis*, and *Fusobacterium varium*	Shotgun metagenomic analysis	Feces	[55]
Lung cancer	Actinobacteria (phyla), *Bifidobacterium* and *Enterococcus* (genus)	16S rRNA gene sequencing	Feces	[56]
